# A Case Report on Dengue Encephalitis With Optic Neuropathy

**DOI:** 10.7759/cureus.9592

**Published:** 2020-08-06

**Authors:** Nawal Khan, Jamil M Bhatti

**Affiliations:** 1 Medicine, Dr. Ziauddin Hospital, Karachi, PAK; 2 Infectious Diseases, The Indus Hospital, Karachi, PAK

**Keywords:** dengue, encephalitis, optic neuropathy, neurologic manifestations, ocular complications, visual loss

## Abstract

The dengue virus is a type of Flavivirus, responsible for causing dengue fever. It mostly prevails in tropical and subtropical countries, with Southeast Asia reporting the greatest disease burden. The virus can affect a multitude of organ systems and the disease spectrum varies from a mild flu-like illness to severe dengue hemorrhagic fever or dengue shock syndrome. Two serotypes, DENV-2 and DENV-3, have been most frequently associated with neurological complications. We report a case of a 19-year-old male presented with signs and symptoms of encephalitis and optic neuropathy, following a diagnosis of dengue fever. Our diagnosis was supported by findings from brain MRI, electroencephalogram, fundoscopy, and a visual evoked potential test. A high-dose intravenous steroid therapy was given in pulses, which resulted in complete visual and neurological recovery. Dengue fever can present with atypical findings due to its propensity to affect multiple organ systems throughout the body. Neurological involvement is not uncommon and hence, clinicians should be aware of such systemic manifestations in order to diagnose promptly. Effective and timely treatment can reduce associated morbidity and result in complete recovery.

## Introduction

Dengue fever is caused by the dengue virus (four serotypes: DENV-1, 2, 3, 4), which is a type of Flavivirus, transmitted through the bite of a female Aedes egypti mosquito. The greatest disease burden lies in Southeast Asia [1]. The subtropical climate of Pakistan makes it susceptible to annual dengue epidemics, the worst of which occurred in 2011 with 22,562 documented cases and 363 deaths [2].

DENV-2 and DENV-3 have been most frequently implicated in neurological complications [3]. Central nervous system (CNS) involvement can be attributed to the neurotropic nature of the virus leading encephalitis, meningitis, and myelitis; the resultant metabolic derangements causing encephalopathy, stroke, or hypokalemic paralysis; or the para- or post-infectious autoimmune reactions responsible for optic neuritis, Guillain-Barre syndrome, and acute disseminated encephalomyelitis [3-7]. The most commonly reported is encephalopathy, comprising of 0.5% to 6.2% of dengue hemorrhagic fever cases [3,5].

We present here a case of dengue fever with neurological involvement leading to optic neuropathy.

## Case presentation

A 19-year-old male from Orangi Town Karachi was admitted to the Ziauddin Hospital on 27 October as a case of dengue fever with four-day history of fever, nausea, vomiting, and generalized abdominal pain. 

On admission, he was vitally stable with an unremarkable physical examination. Biological investigations (complete blood count, urea, creatinine, electrolytes, magnesium, and phosphorus) were normal, except a low platelet count (139 x 10^9^/L) and calcium level (Table 1).

**Table 1:** In-hospital investigations of the patient Hb, hemoglobin; PCV, packed cell volume; TLC, total leukocyte count; Na, sodium; K, potassium; Cl, chloride; HCO_3, _bicarbonate; PT, prothrombin time; APTT, activated partial thromboplastin time; CSF, cerebrospinal fluid; SGPT, serum glutamate pyruvic transaminase; RBC, red blood cell; WBC, white blood cell; GGT, gamma glutamyl transferase; -->, investigation repeated on the same day 12 hours later.

	Dates (2019)					
Parameters	27Oct	28Oct	29Oct	30Oct	31 Nov	1 Nov
Hb (g/dl)	13.6	13.2 --> 13.3	13.9	13.4	12.7	N/A
PCV (%)	42	40 --> 41	43	42	41	38
TLC (x 10^9^/L)	5.1	4.1 --> 3.7	2.7	7.6	5.3	6.3
Neutrophils (%)	65	62 --> 65	69	81	85	79
Lymphocytes (%)	23	24 --> 19	20	10	10	11
Monocytes (%)	11	12 --> 15	11	9	5	10
Basophils (%)	1	2 --> 1	N/A	N/A	N/A	N/A
Platelets (x 10^9^/L)	139	123 --> 112	120	169	207	239
Na (mEq/L)	137	136	138	137	133	136
K (mEq/L)	4.5	4.2	4.5	4.4	4.4	4.2
Cl (mEq/L)	101	100	98	102	98	94
HCO_3 _(mEq/L)	24	24	24	25	27	25
Urea (mg/dl)	36	21	20	28	46	35
Creatinine (mg/dl)	1.03	0.82	0.65	0.61	0.74	0.56
Calcium (mg/dl)		7.54	8.02			
Magnesium (mg/dl)		1.65	2.50			
Phosphorus (mg/dl)		3.13	2.49			
Ammonia (umol/L)				37		
PT(seconds)					11.7	
INR					1.05	
APTT (seconds)					29.2	
CSF analysis	Clear, colorless fluid					
CSF protein (mg/dl)	36					
CSF glucose (mg/dl)	85					
CSF RBC (/cumm)	1					
CSF WBC (/cumm)	0					
CSF gram stain	No microorganism seen					
CSF culture	No bacterial growth after 5 days					
Bilirubin: total	0.98					
Bilirubin: direct	0.18					
SGPT (U/L)	76					
Alkaline phosphatase (U/L)	129					
GGT (IU/L)	68					
RBC folate (ng/ml)			106			
Serum B12 (pg/ml)			593			

**Figure 1:**
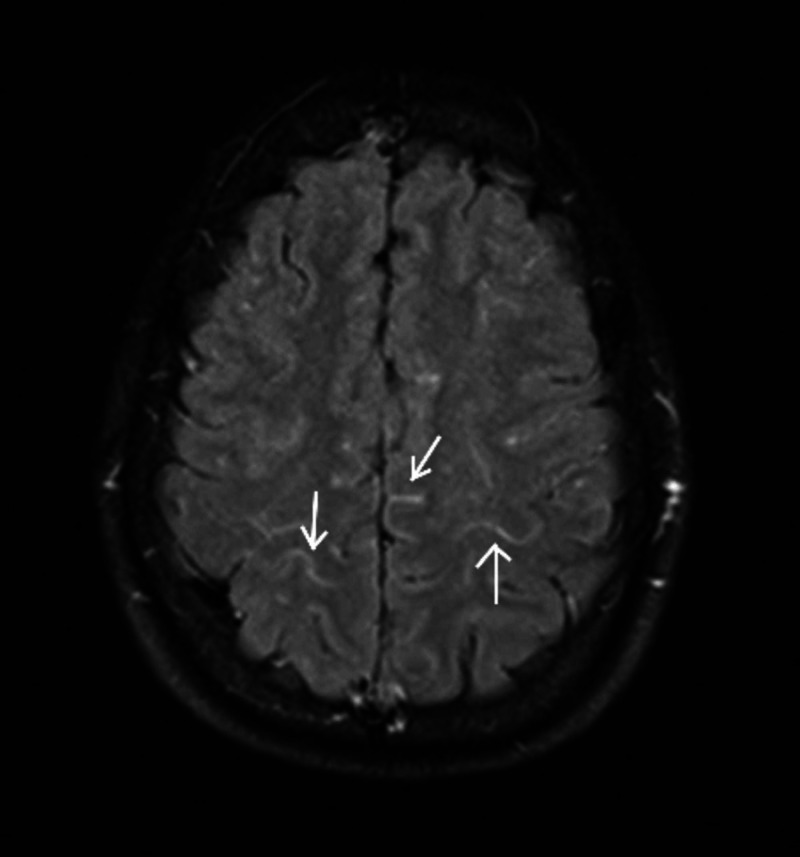
Contrast enhancement seen in cortical sulci of both parieto-occipital lobes.

**Figure 2:**
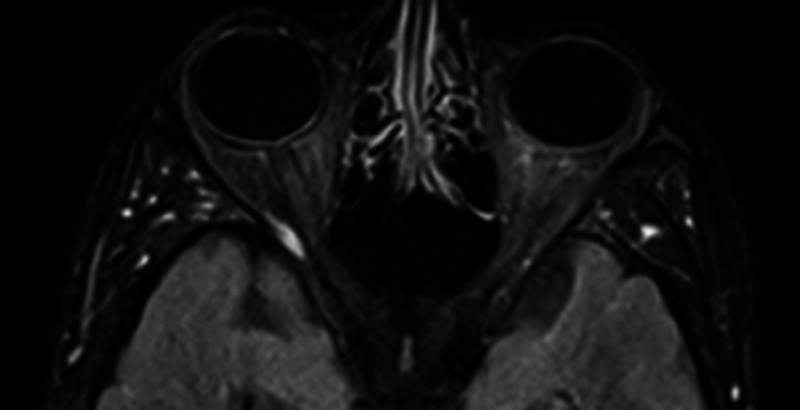
MRI brain fluid-attenuated inversion-recovery (FLAIR) post-contrast image showing normal both orbits and optic nerves, without any significant enhancement.

On second day of admission, the patient reported a sudden onset of bilateral visual loss and generalized weakness. He was found to be irritable with a Glasgow Coma Scale (GCS) of 14/15. He had confused speech and mild nuchal rigidity, but his pupils were reactive to light, and plantars were down going bilaterally. The patient was shifted to the medical ICU, and urgent MRI brain was done that revealed findings suggestive of encephalitis (Figures 1, 2).

A subsequent electroencephalogram confirmed the presence of encephalopathy.

During subsequent days, GCS declined to 13/15. Cerebrospinal fluid (CSF) analysis revealed normal protein and glucose levels, no pleocytosis, and absence of oligoclonal bands. No organism was isolated on gram staining and culture of the CSF. Herpes simplex virus polymerase chain reaction (HSV PCR) was reported negative. Fundoscopy revealed optic neuropathy, which was further validated by the visual evoked potential test. 

GCS further declined over the period of next two days when after consultation with neurologist pulse therapy was initiated in the form of intravenous methylprednisolone for three days with dramatic improvement in clinical condition of the patient was observed. GCS returned to 15/15, and the patient was able to follow commands. The patient was discharged over the periods of next two days but did not come for a follow-up. 

## Discussion

Dengue fever is a vector-borne disease that can manifest in the form of a mild flu illness (fever, headaches, pain behind the eyes, myalgias, arthralgias, nausea, vomiting, and a maculopapular or a macular rash), severe dengue hemorrhagic fever, or dengue shock syndrome, with multisystem involvement.

**Figure 3:**
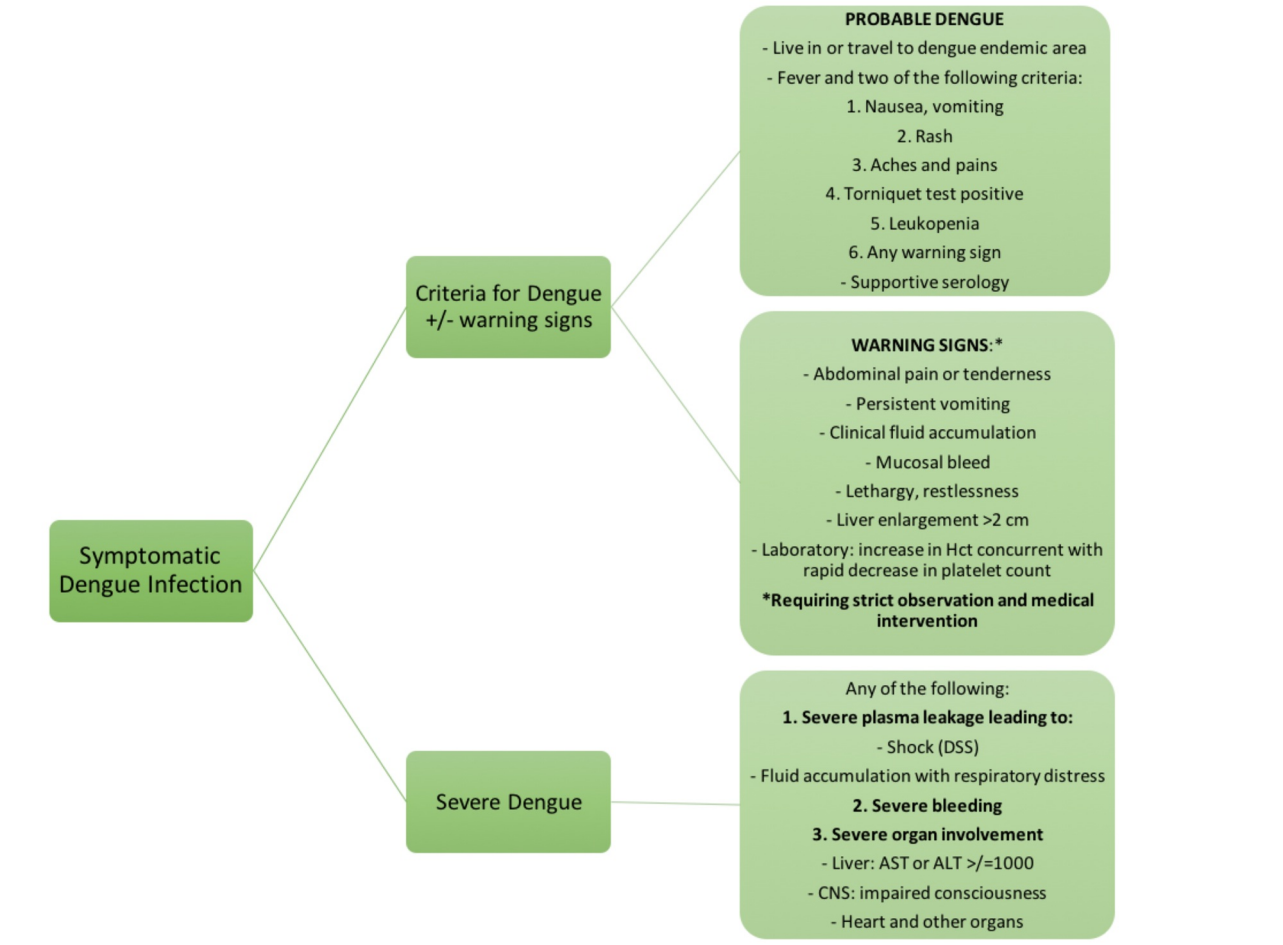
WHO dengue classification ALT, alanine transaminase; AST, aspartate transaminase; DSS, dengue shock syndrome; Hct, hematocrit.

WHO classifies dengue into three categories according to the level of severity: dengue without warning signs, dengue with warning signs, and severe dengue fever (Figure 3) [1]. Dengue can also present with atypical symptoms due to its ability to involve other organ systems. 

The first mention of neurological complications in the context of dengue fever dates back to 1976 [8]. Nervous system involvement occurs in 4%-5% of dengue cases, and while there is no consensus on the underlying pathogenesis, researchers postulate that it may be associated with vasculitis, leaky capillary syndrome with resultant fluid extravasation, direct invasion of the CNS by the dengue virus, metabolic imbalances, or autoimmune reactions [5-7].

The interval between the onset of neurological symptoms and systemic features of dengue fever ranges from three to seven days [6,9]. In our patient, symptoms of encephalopathy and optic involvement manifested five days after the onset of fever.

Patients with encephalitis typically present with fever, headache, altered sensorium, and seizures in the absence of any metabolic imbalances [3,10]. CSF study usually reveals pleocytosis and the virus can be isolated via culture; however, these findings may be absent in some patients, just like in our case [3-5]. In fact, dengue has been identified as the principle cause of encephalitis with normal CSF cellularity (75%) [11]. MRI is preferred over CT scan for neuroimaging, and findings are usually non-specific [5]. Nevertheless, abnormal signal intensities, patchy areas of restricted diffusion, focal hemorrhages, and subtle enhancement after contrast administration have been reported [10]. The basal ganglia and thalamus, followed by the cerebral and cerebellar cortices, are most commonly affected.

Ophthalmic involvement can occur in 40% of the dengue patients, according to a study done in an India during a dengue epidemic [12]. It can involve the anterior or the posterior segment of the eye, manifesting as subconjunctival hemorrhages, uveitis, maculopathy, optic neuropathy, retinal edema, optic disc swelling, vitreous hemorrhage, retinal hemorrhage, or vitritis [5,13]. The time period from the start of fever to the onset of ocular symptoms corresponds to the production of antibodies and deposition of immune complexes and hence, suggests an immune-mediated process as the underlying basis of ocular involvement. However, bleeding tendencies secondary to thrombocytopenia can account for the findings, such as subconjunctival and retinal haemorrhages [14]. Some complications, e.g. uveitis, can present three to five months after resolution of the infection [5].

Supportive management remains the mainstay of treatment of dengue fever, in the absence of no effective antiviral agent. In cases of neurological involvement, adequate hydration, oxygenation, and nutrition should be maintained, consciousness level and hematologic status monitored, and fever controlled with antipyretics [3,13]. In the presence of seizures, standard antiepileptic drugs may be used, and elevated intracranial pressure can be treated with head elevation, mannitol, and steroids. A suspicion of secondary bacterial infection warrants the use of empirical antibiotics [3]. Dengue encephalitis has a good prognostic profile with varying mortality rates from 1.9% to 3.7% [15].

For treatment of ophthalmic complications associated with dengue fever, no definitive treatment protocol exists. The use of high-dose steroids has been reported by several authors due to the presumption that the underlying mechanism is immune-mediated; however, most patients spontaneously recover to their best-corrected visual acuity without any specific treatment [9,12-14]. 

## Conclusions

Dengue usually present with flu-like illness, dengue hemorrhagic fever, or dengue shock syndrome. The clinicians must, however, be aware of diverse manifestations of dengue fever, such as encephalitis with optic neuropathy. Treatment is usually supportive, although sometimes high-dose steroids may be required.
